# Health care workers’ experiences of workplace incidents that posed a risk of patient and worker injury: a critical incident technique analysis

**DOI:** 10.1186/s12913-021-06517-x

**Published:** 2021-05-27

**Authors:** Emma Nilsing Strid, Charlotte Wåhlin, Axel Ros, Susanne Kvarnström

**Affiliations:** 1 University Health Care Research Center, Faculty of Medicine and Health, Örebro University, Örebro, Sweden; 2 Division of Prevention, Rehabilitation and Community Medicine, Occupational and Environmental Medicine Centre, and Department of Health, Medicine and Caring Sciences, Linköping University, Linköping, Sweden; 3 Unit of Intervention and Implementation Research for Worker Health, Institute for Environmental Medicine, Karolinska Institute, Stockholm, Sweden; 4 Region Jönköping County and The Jönköping Academy for Improvement of Health and Welfare, School of Health and Welfare, Jönköping University, Jönköping, Sweden; 5 Region Östergötland, Department of Health, Medicine and Caring Sciences, Linköping University, Linköping, Sweden

**Keywords:** Patient safety, Occupational health, Safety management, Musculoskeletal pain, Psychological distress

## Abstract

**Background:**

Health care workers (HCWs) are at high risk of occupational injuries and approximately 10–15% of patients are affected by an adverse event during their hospital stay. There is scarce scientific literature about how HCWs manage these risks in practice and what support they need. This knowledge is needed to improve safety for patients and HCWs. This study explores HCWs’ experiences of workplace incidents that led to injury or posed a risk of patient and worker injury, with focus on HCWs’ emotions and actions.

**Methods:**

This study employed a qualitative design using the critical incident technique. Semi-structured individual interviews were held with 34 HCWs from three regions in Sweden. Data were analysed using inductive category development.

**Results:**

Altogether 71 workplace incidents were reported. The analysis of two dimensions – the emotions HCWs feel and the actions team members and managers take when a workplace incident occurs – yielded two categories each: Anxiety during the incident, Persistent distress after the incident, Team interplay for safety actions and Support and ratification from managers and colleagues. Health care workers risked their own safety and health to provide patient safety. Teamwork and trustful relationships were critical for patient and worker safety. Support and validation from colleagues and managers were important for closure; unsatisfactory manager response and insufficient opportunities to debrief the incident could lead to persistent negative emotions. Participants described insecurity and fear, sadness over being injured at work, and shame and self-regret when the patient or themselves were injured. When the workplace had not taken the expected action, they felt anger and resignation, often turning into long-term distress.

**Conclusions:**

Work situations leading to injury or risk of patient and worker injury are emotionally distressing for HCWs. Team interplay may facilitate safe and dynamic practices and help HCWs overcome negative emotions. Organizational support is imperative for individual closure. For safety in health care, employers need to develop strategies for active management of risks, avoiding injuries and providing support after an injury.

**Supplementary Information:**

The online version contains supplementary material available at 10.1186/s12913-021-06517-x.

## Background

In Western countries, injury rates are higher among health care workers (HCWs) than among workers in any other field [1]. Being in patient care, HCWs are exposed to several occupational health hazards, such as viruses [2], ergonomic or physical hazards [3, 4] and physical violence [5]. Threats and violence are in fact more frequently reported in Swedish health care than in other Swedish workplaces, and the percentage of those exposed has increased in the last years [6]. All of these exposures may lead to a variety of injuries such as musculoskeletal injury, wounds, infections or psychological symptoms [7, 8]. The risk factors for occupational injury can be attributed to an interaction between characteristics of the worker and the patient, and the workplace [9, 10]. Adverse events may affect not only HCWs but also patients, and in addition may have a negative impact on relatives and health care organizations, all considered as a significant problem [11, 12]. Approximately 10–15% of patients are affected by an adverse event during their hospital stay, and a substantial part of these events are considered preventable [13, 14].

A few studies have explored the relationship between working conditions and both patient and HCW injuries. The findings show that a high safety climate supports safe patient care and also ensures HCW safety [15–18]. Pousette and co-authors (2017) concluded that safety improvement interventions should be planned so that both patient safety and occupational safety and health are considered concomitantly, and not separately in different processes as has been previously done in practice and research [15].

Qualitative study designs are used to explore the experiences of individuals, information that is difficult to obtain with quantitative methods [19]. Recent literature has highlighted that adverse events affecting the patient, the first victim, are also stressful for the HCWs involved, who are therefore commonly considered as secondary victims [20]. In a recent systematic review and meta-analysis, more than two-thirds of the affected HCWs reported troubling memories, anxiety, anger, remorse and distress [21], and elsewhere it was shown that the support received influences how the worker will feel about the error as well as the recovery process [21–23]. Despite the amount of literature about occupational hazards in health care, there are few studies on how HCWs experience work situations where they have been injured [24]. To the authors’ knowledge, there are no previous studies exploring HCWs’ experiences of situations at work putting them and patients at risk of injury.

To improve safety at work and reduce harm for both patients and HCWs, it has been suggested that we should shift our focus from creating absolute safety, i.e. from the unattainable goal of zero harm, to doing a better job of actively managing risk [25]. This active management of risk requires engagement and actions at all managerial levels as well as coordinated actions between managers and frontline staff [25]. As suggested in “resilient health care” studies, safety can be described as the capacity of a system (its individuals, teams and organization) to adapt to varying conditions such as threats, hazards, possibilities and a changing work environment, so that the system succeeds in its activities and tasks [26]. In order to develop dynamic, practical strategies, we need descriptive studies that will help to identify degraded conditions and the risks they pose to patients and HCWs [25]. There is scarce scientific literature about how HCWs manage these workplace risks in practice and what support they need. This knowledge is needed to further improve the safety of patients as well as occupational safety and health for HCWs.

### Aim

The aim of this study was to explore HCWs’ experiences of workplace incidents that led to injury or posed risk for patient and worker injury, with a special focus on their emotions and actions.

## Materials and methods

### Design

This study employed a qualitative design using the critical incident technique (CIT) [27] to explore HCWs’ experiences of workplace incidents. “Health care workers” is used as an umbrella term referring to people involved in promoting, protecting, caring for or improving the health of the population [28].

Individual interviews were used for data collection. The technique in CIT consists of a set of procedures for gathering observations of human behaviour in defined situations [27]. Incidents may be events, activities or role behaviours that affect an outcome and are memorable to those involved [29]. To be critical, an incident must occur in a situation where the purpose or intent of the act seems fairly clear to the observer and where its consequences are sufficiently definitive to leave little doubt as to its effects [27]. The method implies that distinct questions are asked, which enables participants to describe their experiences of critical incidents at work, as well as their thoughts, actions and processes during the situation [29]. CIT originates from the 1950s, but the approach is still applied in contemporary qualitative studies in health care contexts [30, 31]. Compared with other inductive approaches, the CIT approach was chosen due to the potentials to explore HCWs experiences of specific workplace incidents as the unit of analysis [30], in line with the research aim.

### Setting and participants

This study was conducted in three health care regions in central Sweden with a total of nine hospitals and about 120 health care centres. Information about the study was sent to the regions’ human resources departments, health care departments and trade union departments, which further distributed the information to health professionals. Information about the study was also distributed through social media channels, the unions and the regions’ internal websites. Interested HCWs and heads of health care departments contacted the project manager (C.W.) for more information about the study. The information stated that participation was voluntary and could be discontinued at any time without explanation, and that all data would be handled confidentially. One of the main researchers (E.N.S. or S.K.) contacted the HCWs who had agreed to participate. Inclusion criteria were: HCWs who had experience of at least one workplace incident with risk of injury for both the patient and themselves and ability to understand Swedish. We aimed for a range of experience and purposeful sampling in terms of age, gender and profession was applied. Thirty-seven individual interviews were held between April 2016 and February 2018. Two interviews were excluded because of technical problems during recording and one because the incident reported had occurred too long ago.

In total, 34 interviews were included in the analysis. Mean age of the HCWs was 46 years and the majority were registered nurses (Table 1). The settings where the incidents occurred were health care centres and hospital departments including emergency care, geriatric care, intensive care, internal medicine, primary health, psychiatric care, radiology, audiology, rehabilitation medicine and surgery.

**Table 1:** Characteristics of the participants

***Sex***	
Female	23
Male	11
Total	34
***Mean age (range), yrs***^**a**^	46.5 (21–64)
***Profession***	
Registered nurse	13
Certified nurse assistant	7
Nurses’ aide	6
Registered physiotherapist	4
Physician, psychologist, or other	4
***Number of years in the profession***^**a**^	
Mean	19
1–5	5
6–10	5
11–20	7
> 21	16

^a^information missing on one informant

### Data collection

Semi-structured interviews were performed based on an interview guide. The interview guide was based on a guide for researchers using the CIT [29], but developed by the research team for this study and is provided as Additional file 1. A pilot interview was conducted to evaluate the questions and to refine and coordinate the researchers’ interview technique and the procedures, since two researchers (E.N.S. and S.K.) would be holding the interviews. Only minor revisions were made to the interview guide and the pilot interview was therefore included in the final sample. The interview started with the main question: “Describe a work situation (a critical incident) when you experienced injury risk for both you and your patient.” This question was followed by more specific questions regarding the context, the participating HCWs’ own actions, thoughts and feelings, actions of others, how the situation was resolved, and how the participant and the patient were affected by the situation. Probing questions were used to develop and deepen the answers. The role of the interviewer was to enable the participants to be as specific as possible in their description of the critical incident [29].

A time and place for the interview was set in agreement with each participant. Written informed consent was obtained before every interview. The interviews were held in a private room at the participant’s workplace or the researcher’s office. All 34 interviews were face-to-face interviews except three, which for reasons of illness or convenience had to be performed as telephone interviews. Each interview lasted between 25 and 66 min, mean 49 min. The interviews were digitally recorded and transcribed verbatim by a professional transcriber. To preserve confidentiality, participants were pseudonymised and each assigned a code name.

### Data analysis

An inductive category development was used as described by Flanagan [27]. NVivo 11 and 12 was used to manage and code the data (QSR International, Melbourne, Australia). All the transcripts were read to get a sense of the whole and to discover similarities and differences. Two transcribed interviews were jointly analysed by three researchers (E.N.S., S.K. and C.W.) to develop a tentative coding scheme. Two of the researchers (E.N.S. and S.K.) coded all the transcripts and continuing discussions were held throughout all analytical steps. First, incidents related to the aim were identified and subjected to structural analysis aiming to describe the type of incident (Table 3). In the second step, meaningful units of the experiences of incidents were derived, coded and divided into actions and emotions. In a third step, these codes were compared to find similarities and differences and with the aim of grouping them together into sub-categories, and thereafter into categories and, finally, main areas. The purpose of a category is to describe the general character of the sub-categories, while that of a main area is to describe the overall theme contained in the data. Examples of the coding strategy is provided in Table 2. This analytical process is similar to that described in previous studies of HCWs’ experiences, emotions and actions using CIT [30, 32–34]. There is no consensus on a definition of the term “emotion”, and the scientific use of the term reflects several different meanings of “emotions”. In this study, we acknowledge that “emotion” consists of neural circuits, response systems, and a feeling state/process that motivates and organizes cognition and action, as previously described [35].

**Table 2:** Examples of coding strategy

Quotation	Code	Sub-category	Category	Main area
Disgusting and you feel all the time this, why didn’t I do anything, why didn’t I do more (Fia)	It was disgusting. Why didn’t I do more.	Shame and self-regret when a patient had been exposed to risk of injury	Persistent distress after the incident	Emotions evoked by an incident
I thought it was I who caused it, as I didn’t walk in to his room when he was at the toilet, could I have avoid it (Vera)	I caused it or I could have avoid it			
Permanent, actually. It (the pain) has impacted on my whole life (Greta)	Pain impacts on the whole life	Sadness over being injured at work		
I can’t say it’s a success story because I would rather be without it, so to speak. It still hurts when I do something stupid (Frida)	Incident no success story. Still suffering from pain.			

During the analytical process, tentative categories were modified and redefined, and new categories were developed where needed. The categories were aimed to be internally homogeneous and externally heterogeneous but when a behaviour fitted into more than one sub-category, the category that best fit the described behaviour was chosen. To strengthen the confirmability, the two authors who were responsible for the data analysis (E.N.S. and S.K.) held continuous consensus discussions throughout the analysis until agreement upon final sub-categories, categories and main areas was reached. A third author (C.W.) reviewed the adequacy of the categories and areas derived from the data analysis. Finally, all authors discussed the categorization and agreed upon the final version. Quotes capturing the essence of what was said were selected to illustrate the different categories. The selected quotes from the transcripts were translated into English and then retranslated into Swedish, to ensure that their meaning was retained.

Permission to conduct the study was obtained from the regional ethical board in Linköping (dnr 2015/330–31 and 2016/197–32). The participants provided written informed consent after receiving both written and oral information about the study, including the voluntary nature of participation, the possibility to withdraw at any time without explanation, and assurance that all collected data would be handled confidentially and no individual would be identifiable in the quotes or the results. Only the research team had access to the original interview files, transcripts and informed consents. The participants were also informed of the interviewer’s professional background, reasons for interest in the topic and that the interview data would be analysed and published in a research journal. The study followed the Consolidated Criteria for Reporting Qualitative Research (COREQ) checklist [36].

## Results

In total, 71 workplace incidents were identified in the 34 interviews (Table 3). Every participant described at least one incident (range 1–5). HCWs’ experiences of workplace incidents that led to injury or posed risk for injury that emerged from the analysis comprised two main areas with two categories and four to five sub-categories each. The first main area Emotions evoked by an incident comprised the categories: Anxiety during the incident and Persistent distress after the incident. The second main area Actions by team members and managers covered the categories: Team interplay for safety actions and Support and ratification from managers and colleagues (Table 4).

**Table 3:** Description of the reported critical workplace incidents posing a risk for the patient and the health care worker (HCW), categorized by type of situation

***Type of critical incident*** *(71)*	
Violence or threat (37)	
- Patient threatening or violent (27)	
- Close relative threatening or violent (5)	
- Other threatening situation (5)	
Moving and manual handling of patients (30)	
- Patient falls or nearly falls (20)	
- Equipment and external environment (6)	
- Other situation (4)

**Table 4:** Summary of main areas, categories and sub-categories regarding health care workers’ (HCWs) experiences of workplace incidents that led to injury or posed risk for patient and worker injury

Main area	Category	Sub-category
Emotions evoked by an incident	Anxiety during the incident	To feel safe within the team
		Feelings of insecurity
	Persistent distress after the incident	Shame and self-regret when a patient had been exposed to risk of injury
		Sadness over being injured at work
		Anger and resignation when managers had not taken necessary action
Actions by the team and managers when handling an incident	Team interplay for safety actions	Act adequately and supportively
		Take responsibility and team leadership
	Support and ratification from managers and colleagues	Informal debriefings with colleagues to release emotions
		A validating approach and follow-ups by the manager

### Emotions evoked by an incident

The main area describing the emotions HCWs experience when handling a work situation that led to injury or posed risk for worker and patient injury resulted in two categories: Anxiety during the incident; and Persistent distress after the incident.

#### Anxiety during the incident

This first category is supported by two sub-categories, To feel safe within the team; and Feelings of insecurity, and elucidates the emotions HCWs expressed when recalling a workplace incident posing risk of injury to themselves and a patient. The HCWs often described satisfaction with team actions during an incident and expressed pride when no one had been injured. They highly valued working with experienced and trusted colleagues as it made them feel safe and as it contributed to a safer workplace. Previous experiences of incidents and risks at work made the HCWs feel prepared for incidents to happen. Working in critical settings such as psychiatric care or at emergency departments was described as a challenge but could also make HCWs feel blunted. Safeness was characterized by trust in the team and having an open and friendly atmosphere without judgements.

*Of course you have to be able to say, “Yeah but, I … I can do this. I’m not afraid,” or “Whoa, I don’t want to go in to see him alone.” You have to decide for yourself what does and what doesn’t feel okay, and not just pretend something is okay. (Klas)*

Feelings of insecurity in unsafe situations, especially in sudden, unexpected situations, were described by the HCWs. These could involve anxiety and fear of making mistakes or acting wrong – also, fear of what others, such as the relatives of an injured patient, might think. Not knowing how to handle a threatening situation was connected to feelings of stress and even panic; sometimes, it damaged the self-image. Most prominent were descriptions of fear of what a threatening or violent patient was capable of, fear of being hurt during the incident and of what the consequences might be.

*Oh yeah, there was one small, horrible thing, because I was really afraid that I would wreck my back when I was to turn 65 and retire, that wrecking my back would be the last thing I did. So my thought was this must not happen, and it was not a big deal, so everything’s okay, but of course you don’t know that then when it happens… (Vera)*

According to the HCWs, when they felt focused during the actions, they were able to regulate their emotions, and could act as required and feel competent, even though they might feel afraid and though they worried initially. They unloaded their feelings and emotions afterwards.

#### Persistent distress after the incident

The second category conveys emotions of distress that the HCWs still felt a long time after an incident had happened. The category consists of three sub-categories: Shame and self-regret when a patient had been exposed to risk of injury; Sadness over being injured at work; and Anger and resignation when managers had not taken necessary action. Feelings of blame and self-regret were recurrent throughout the data regardless of setting or type of incident. The HCWs wondered whether they had acted correctly, what they should have done instead, and how the incident could have been avoided. They described ambivalence about what would have been the correct way to act. An incident such as a patient fall, described as difficult to predict, was acknowledged as a failure even when all necessary actions had been taken to prevent it. Some participants described these incidents as “just bad luck”. Resignation over workplace actions such as use of different work equipment for handling patients, which sometimes turned out to constitute a risk for both patient and HCW, was described. It was evident that some participants felt ashamed and guilty about having caused an incident. The HCWs expressed recurrent thoughts of self-regret, sometimes several years after an incident, as illustrated in the quotation below.

*Did that cause me to be more vulnerable afterwards? Would it have been worth it, then, not to have done that? And that it would have been better for me in the long term... and that my life, you know, if I had chosen not to do that ... – that thought has occurred to me … (David)*

The HCWs provided a substantial number and variety of descriptions of pain located in the neck, back, shoulder, arm, hand, hip, and foot after being injured at work. Mostly, they had returned to work immediately or after a few days off, but in some cases, the acute pain developed into disability and long-term sickness absence. The HCWs were emotionally affected when talking about an incident that had happened and especially about the consequences the incident had had, and continued to have, for their working and private life. They kept struggling with memories, emotions of worry and stress reactions that were often evoked when they were exposed to noise, places or persons reminding them of the incident. Some HCWs described having sacrificed their own health for the sake of a patient, and in some cases they related that the patient had not noticed this or had died anyway. This created feelings of emptiness. Overall, sadness was the overarching emotion.

*I felt it was a pity; it was a failed mobilization that frightened the patient, which reduces the outlook for future mobilization and, well, ultimately puts my health at risk, too. (Albert)*

*Lasting, actually. That [injury] has affected my entire life situation, you know? (Greta)*

The third and last sub-category, Anger or resignation when managers had not taken necessary action, conveys the emotions HCWs expressed when describing how the incident had been handled at the workplace by the manager. The sub-category also includes feelings of anger evoked by being hit by a patient they were treating. Some of the HCWs related that their manager had made some adjustments at work or helped them to change to another department after an incident. The perceived support from the managers and the emotions this evoked varied substantially. The HCWs expressed anger when, previous to the incident, they had informed their manager of risks at work, but the manager had not taken any action or had not responded sufficiently, and the anticipated incident had then happened. Such risks for patient injury included lack of a care plan, lack of overall medical responsibility, lack of routines or poor quality of equipment. Risks related to the psychosocial work environment, such as high workload, were described as risks for both patient and HCW injury. Incident reports were written, but when actions and feedback from the managers were perceived as deficient, the HCWs stopped writing reports. Often, they felt not listened to, and they felt sad, angry and dejected when they perceived negligence from the managers. In the quotation below, a physician expresses anger at having previously complained about poor quality of material and not having obtained the desired response. When she was injured as a result of the problem that she had identified, she did not get an appropriate response either.

*I can still feel it was a real bummer to have to pay for that sick day out of my own pocket. I’d say that’s almost a slap in the face! Now, I’m not going to go bankrupt just because I lost a day of work, but that’s not the thing that really bothers me; it’s the attitude! Here we’ve identified a problem, and then an incident occurs as a result of the problem – it was not unknown. (Cia)*

### Actions by the team and managers when handling an incident

The main area describing HCWs’ actions when experiencing a situation at work that led to injury or posed a risk for worker and patient injury includes the two categories: Team interplay for safety actions; and Support and ratification from managers and colleagues. This main area incorporates actions taken by the team during an incident as well as the actions taken by colleagues and managers after such an incident has happened.

#### Team interplay for safety actions

This category includes two sub-categories: Act adequately and supportively; and Take responsibility and team leadership. Under the first sub-category, Act adequately and supportively, the HCWs described themselves as being well coordinated and assisting each other. Clinical decisions, for example regarding patient manual handling, were based on updated information on patient health and functioning, and were made in mutual agreement with colleagues, including decisions to discontinue a mobilization effort. The HCWs described being aware of their own work postures and safety. They said that colleagues acted quickly when needed, and gave examples such as colleagues coming running to assist in a near fall situation or taking physical control over a violent or confused patient. Violence and threats were present in all clinical settings. These situations were described as often unpredictable and when organizations and HCWs were not prepared, individual initiatives were taken to avert the threat. The HCWs put trust in their colleagues’ competence. Team interplay and trustful relationships within the team were highlighted as important for safe actions and good outcomes for both the HCW and the patient. The participants described that, before acting in an emergency situation, they made an assessment based on clinical reasoning, not only of the situation itself, but also of the patient and the colleague they were working with. The quotation below illustrates how an assistant nurse shaped his perception of the situation and the actors involved, before acting.

*What happened? Who’s that lying there? Who – what patient is that? [...] And who’s holding the patient? Who am I supposed to be working with? (Klas)*

There were also situations when team interplay failed, such as performing patient manual handling alone, instead of waiting for other team members and despite acknowledging the risks. The HCWs described a focus on the patient with little thought for their own ergonomics or injury risk, especially in falls or near falls, but also in other situations such as when performing cardiopulmonary resuscitation:

*You just had to get on with it. It was like ... I didn’t think about myself at all, that anything could go wrong. I was focusing only on the patient. (Wilma)*

The second sub-category, Take responsibility and team leadership, includes the importance of someone taking the main responsibility, making decisions and guiding colleagues. Giving positive feedback and praising each other was also important for team interplay. According to the participants, the team leader was normally the person who was the most skilled and experienced, and who was highly trusted by the team. Planning was crucial for safe actions and the team should clarify the responsibility before actions took place; if they did not do this, safety could be jeopardized, as illustrated in the quotation below where a patient had a near fall.

*… the problem was the allocation of responsibility, since this CNA [certified nursing assistant], who was experienced, was in the room, and I was put in as an extra resource [...] and she had prepared everything and kind of wanted to be in charge. So the roles were mixed up from the start, so I somehow didn’t have it in me to stand my ground and demand an additional resource before we started, and during the mobilization she took over more than what I had intended. (Albert)*

#### Support and ratification from managers and colleagues

The second category, Support and ratification from managers and colleagues, includes two sub-categories: Informal debriefings with colleagues to release emotions; and A validating approach and follow-ups by the manager. To discuss an incident with a colleague directly after the incident was described as important. Experiences were shared and discussed in a friendly atmosphere where support and encouragement were given and the HCWs were able to somehow lessen the gravity of the situation and laugh together. These discussions represented informal debriefings in the team and released negative emotions.

*… so it was my co-worker, another nurses’ aide, and I who talked about it, since we’d both seen what happened and we’d had the same reaction, you know. But we talked about it a lot afterwards and that was helpful ... also with other staff, and so on. So that was a way to put it all behind me. (Lena)*

However, there were also descriptions of unwillingness at the workplace to discuss difficulties. Reasons for not bringing incidents up in discussions were respect to a patient, heavy workload, and stress; but sometimes there was also a reluctance to uncover and deal with problems.

The second sub-category, A validating approach and follow-ups by the manager, deals with how managers’ attitudes and actions affect how HCWs experience an incident and its consequences. A listening and emphatic manager, who follows up the incident, encourages writing an incident report and modifies work tasks when needed, was described as supportive. This approach was perceived as a validation of the HCW’s experience as it creates feelings of trust and conveys a sense of ratification, which may be important for how an HCW may feel about and cope with an incident and its consequences.

*I stuck to my guns and the boss listened to me. Then we sat down and had a good chat with the medical supervisor and with everyone who had been involved and the boss. And also, for a long time I’ve been pushing the idea that we have to have a routine [...], so I can feel safe at work. (Erika)*

There were also descriptions of unsupportive management, for example involving managers being hard, not listening, not showing understanding for the HCW’s experience, or not giving feedback as expected. Some HCWs were told that, as an HCW in a certain work context, “one should expect risks” for occupational injury and then go on: business as usual. Absent managers, that is, managers who were not present and did not regularly meet with their HCWs, were described as problematic as they showed a lack of understanding of the work situation. An unsatisfactory response from the manager and insufficient opportunities to debrief an incident and the emotions it evoked could shape negative emotions.

*You might not always have the supervisors’ understanding that it does involve such a risk factor, instead, well, you’ll have to make sure they [the patients] sit still then, but that’s not always easy. (Fia)*

## Discussion

The aim of this study was to explore HCWs’ experiences of workplace incidents that led to injury or posed risk for patient and worker injury, with a special focus on their emotions and actions. The novelty revealed in this analysis is that work situations leading to injury or posing risk for patient and HCW injury are emotionally distressing for HCWs. Team interplay may facilitate safe and dynamic practice and help HCWs to overcome negative emotions, but organizational work support is imperative for individual closure. Our analysis underscores the need for employers to develop strategies together with HCWs and teams for active risk management at work, to avoid injuries and provide support after an injury, regardless of whether it was the patient or the worker who was at risk or injured. That organizational support provided by colleagues and managers was perceived as important is in line with the theory of perceived organizational support, which concerns employees’ general belief that their work organization values their contribution and cares about their wellbeing [37–39]. Perceived organizational support is suggested to be especially helpful in reducing traumatic consequences of stressors at work [37]. Feeling safe within the team, which incorporates trustful relationships, team leadership, confidence in each other’s competence, and openness to share emotions, was described as important for safe and dynamic practice. To share emotions means also that somebody is listening, which is an example of empathic support provided by colleagues that may be important for the HCW to cope with the incident. These are findings that can be seen as part of the organizational culture of HCWs’ workplaces, which can be related to safety for patients and HCWs [40]. The findings also emphasize the importance of good teamwork for active risk assessment and management of risks. Such teamwork requires engagement, mutual understanding and coordinated actions between managers and HCWs as well as within the team, both during and after an incident, as suggested for resilient health care [25]. Resilience has been suggested to depend on four capacities in a system, which are: to be able to react when something happens; to monitor what is happening; to anticipate what might happen; and to learn from everything that happens [41]. To achieve better safety in a system, efforts should be taken to increase these capacities. In this study the HCWs gave examples of these capacities, such as being able to react as an individual and a team when something happens; to monitor what has happened and write incident reports (as well as expect feedback and reactions); and to anticipate what might happen by planning work tasks, following routines and being part of a trustful and competent team. Finally, managers capable of giving feedback, reacting and providing support are needed to enable organizational learning and individual closure. The findings of this study point to possibilities for further strengthening of both patient safety and occupational safety and health. One of the core strategies for patient and HCW safety may be to develop and maintain good teams with sufficient resources, as well as to provide opportunities for the teams to reflect on risks. Our study identified the importance of good teamwork for safety and in this respect our findings share some similarities with the findings from previous qualitative studies describing what HCWs perceive as important for patient and worker safety [42–44]. In a focus group study with nurses and physicians, patient and worker safety was found to be determined by interpersonal communication with colleagues, trust and worker readiness to take responsibility, as well as emotional and practical support [42]. The HCWs in our study prioritized protecting the patient, sometimes at the expense of their own safety, as also described elsewhere [43]. Our analysis revealed conflicting actions and attitudes, for example when HCWs caught a falling patient and performed patient handling alone or with too few personnel, despite the inherent risk. This could be interpreted as risk acceptance. When team members do not respect or agree to a request for an extra person in patient handling, this could be devastating for both teamwork and the safety of the patient and worker; also, it can create feelings of powerlessness and self-regret of the injured worker. Respect for its individual members is important for a team in order for it to be effective, as previously demonstrated [44], and our findings underscore the need to disclose teams’ values and norms as risk acceptance may be negative for both patient and worker safety.

In some situations, HCWs may have to physically control the patient, using different physical restraint methods and devices. These devices, in turn, may increase the risk of patient injury and extend the duration of the hospital stay [45]. These complicated events create persistent emotions of distress and present HCWs with ethical dilemmas, which the present study contributes to explore. In one study, perceived high team efficacy in dealing with patient and visitor aggression was shown to be predicted by physical environment, a shared positive organizational attitude and psychiatric setting [46]. The authors conclude that general hospitals could benefit from approaches in psychiatry focusing on staff efficacy in the management of patient and visitor aggression. Managers may need support or training for this task by their organizations.

Our results emphasize that workplace incidents posing risk for patient and worker injury are emotionally distressing for HCWs and that self-regret and distress can last for a long time if not properly addressed. Perceived organizational support may influence how HCWs feel about the recovery process. In this study, we show that the absence of a safety culture to reflect on risks and incidents and insufficient manager support could create emotions of anger and resignation that could last for many years. The experiences of HCWs as second victims of error when a patient has been injured have been explored and synthesized in a few recently published systematic reviews [21, 23]. The findings indicate that an error brings a considerable and longstanding emotional burden to the HCW [21, 23]. Second victims have been shown to experience negative emotions after an adverse event such as anxiety or anger directed towards themselves, towards others or to the work environment [21, 22] and it has been reported that an error can have a negative impact on self-image and interpersonal relationships at the workplace [23]. The findings from our study emphasize that active support is required to reduce negative emotional responses and to facilitate coping after an incident and create a just culture free from blame or judgment. This active support should include direct and emphatic collegial and managerial support, follow-ups and provision of psychological support when needed. To regularly reflect on incidents and risks within the team is another important strategy that may be facilitated by local managers. It has been suggested that supporting all victims of patient harm (i.e. patients, their relatives, and HCWs is one important strategy in patient safety management [47].

### Methodological considerations

The limitations and strengths of this study are discussed in terms of the components of trustworthiness: credibility, dependability, confirmability and transferability [30, 48]. The CIT [27] was employed to explore HCWs’ experiences of workplace incidents. CIT is widely used in contemporary qualitative research to explore the perspective of patients or healthcare providers [29–34]. As previously described when using CIT in qualitative research [30], the data collection was based on the individual perspective and the interviewees were encouraged to recall real incidents with a clear beginning, clear end and generating actions that had an impact (positive or negative results). This detailed description of the incidents provided by the participants is considered a strength of CIT and this study, as it can be assumed that this information is accurate, strengthening credibility. The participants in this study reported 71 unique critical incidents, which is within the recommendations of 50–100 critical incidents required to describe a problem [27]. The purposeful sampling strategy strived for a wide range of experiences and resulted in a diverse variation of descriptions of workplace incidents that occurred in different parts of the health care system. This can be interpreted as a strength, but may also restrict the transferability. The authors responsible for data collection and analysis (E.N.S. and S.K.) had previous experience in conducting qualitative studies. These two authors held the interviews and individual differences may have influenced the data, but dependability was strengthened through efforts to fine-tune and coordinate the researchers’ interview techniques and procedures. There are both advantages and disadvantages to interviewing one’s peers. To promote transparency, the participants were informed of the interviewer’s professional background. As a fellow practitioner, the participants’ confidence may be gained more readily than if the interviewer were a non-practitioner, and the shared knowledge and interest between the participants may increase the interviewer’s credibility. Although the participants were encouraged to openly convey their experiences, there may always be a professional vulnerability and risk of feeling judged [49]. There was no relationship prior to the interviews. All of the authors were researchers with several years of health care experience, working as a nurse, physician, and physiotherapist, as well as a human resources manager and consultant in patient safety. The authors had good knowledge of CIT, occupational safety and health, and patient safety. These different theoretical and practical perspectives may have enhanced the understanding of the phenomenon of workplace incidents. We have been aware of our perspectives during the data collection and analysis, and have strived to correctly convey the participants’ perspectives in the results. The data were analysed systematically and independently using inductive category development as previously described [27, 30]. To strengthen confirmability, the entire research team held consensus discussions throughout the analysis and the findings were finally approved by the research team and discussed with other researchers and HCWs. Confirmability and transferability were reached by providing a description of the participants and setting, enabling the reader to decide whether these findings can be transferred to other, similar contexts. It was not within scope of this study, but to collect and analyze multiple perspective, using triangulation of diverse data sources such as to combine incident reports with interviews with HCWs, managers and patients could have given more information about the context and enriched the data.

The findings from this study may shed light on HWS’ journey after their experience of a workplace incident, which can be long and distressful. These findings may serve as a starting point for further research on how to develop strong health care teams that can perform active risk assessment and manage risks built on trustful relationships and support by managers. The clinical implications of these results indicate a need for increased focus, among managers, on occupational safety and health at the workplace and highlight the importance of manager strategies to validate the HCW’s experiences, take actions to prevent recurrence of safety breach incidents, strengthen teamwork and provide tools for collegial support and feedback.

## Conclusions

The key finding in this explorative study is that work situations leading to injury or posing risk for patient and HCW injury are emotionally distressing for HCWs. Team interplay is critical and may facilitate safe and dynamic practices and help HCWs to overcome negative emotions, but organizational support is also imperative for individual closure. For safer health care for both patients and workers, there is a need for employers, together with employees, to develop strategies for active management of risks, avoiding injuries and providing support after an injury.

## Supplementary Information

**Additional file 1.**

## Data Availability

The data that support the findings of this study are available on request from the corresponding author [E.N.S.]. The data are not publicly available due to them containing information that could compromise research participant privacy/consent.

## Abbreviations

CIT: Critical incident technique
HCWs: Health care workers
