# Supplementary material for: Health care workers’ experiences of workplace incidents that posed a risk of patient and worker injury: a critical incident technique analysis
Source: BMC Health Serv Res. 2021 May 27;21:511. doi: 10.1186/s12913-021-06517-x (PMC8157721; doi:10.1186/s12913-021-06517-x)
Supplement: Supplementary file 1 — Additional file 1. [file 12913_2021_6517_MOESM1_ESM.docx]

# Interview guide

1. Please tell me about a situation where you and a patient were injured, or at risk of being injured. Be as specific as possible, from the start of the incident until it was over.
   1. What happened? (where were you, how did it start)
   2. What did you do (what did you think, feel)
   3. If there were others there, what did they do?
   4. How was the incident solved?
   5. How has this incident affected you, the patient or other?
   6. What happened then?
   7. How do you think the incident could have been avoided?
2. Have you experienced another incident that you would like to tell me? If so, the interview guide follows the steps 1a-g,
3. To conclude: Is there anything you would like to clarify or discuss that we have not discussed today?
